# Peptide Derivatives of Platelet-Derived Growth Factor Receptor Alpha Inhibit Cell-Associated Spread of Human Cytomegalovirus

**DOI:** 10.3390/v13091780

**Published:** 2021-09-06

**Authors:** Berenike Braun, Dina Fischer, Kerstin Laib Sampaio, Maja Mezger, Dagmar Stöhr, Richard James Stanton, Christian Sinzger

**Affiliations:** 1 Institute for Virology, Ulm University Medical Center, 89081 Ulm, Germany; berenike.braun@uni-ulm.de (B.B.); dinafischer@gmx.net (D.F.); kerstin.laib@uni-ulm.de (K.L.S.); maja.mezger@uni-ulm.de (M.M.); dagmar.stoehr@uniklinik-ulm.de (D.S.); 2 Division of Infection and Immunity, School of Medicine, Cardiff University, Cardiff CF14 4XN, UK; stantonrj@cardiff.ac.uk

**Keywords:** human cytomegalovirus, clinical isolates, cell-associated spread, cell-to-cell spread, multiplicity of infection, PDGFRα-peptides, entry inhibitors

## Abstract

Cell-free human cytomegalovirus (HCMV) can be inhibited by a soluble form of the cellular HCMV-receptor PDGFRα, resembling neutralization by antibodies. The cell-associated growth of recent HCMV isolates, however, is resistant against antibodies. We investigated whether PDGFRα-derivatives can inhibit this transmission mode. A protein containing the extracellular PDGFRα-domain and 40-mer peptides derived therefrom were tested regarding the inhibition of the cell-associated HCMV strain Merlin-pAL1502, hits were validated with recent isolates, and the most effective peptide was modified to increase its potency. The modified peptide was further analyzed regarding its mode of action on the virion level. While full-length PDGFRα failed to inhibit HCMV isolates, three peptides significantly reduced virus growth. A 30-mer version of the lead peptide (GD30) proved even more effective against the cell-free virus, and this effect was HCMV-specific and depended on the viral glycoprotein O. In cell-associated spread, GD30 reduced both the number of transferred particles and their penetration. This effect was reversible after peptide removal, which allowed the synchronized analysis of particle transfer, showing that two virions per hour were transferred to neighboring cells and one virion was sufficient for infection. In conclusion, PDGFRα-derived peptides are novel inhibitors of the cell-associated spread of HCMV and facilitate the investigation of this transmission mode.

## 1. Introduction

Human cytomegalovirus (HCMV) is a ubiquitously distributed member of the herpesvirus family. In most cases, the infection is not noticed in immunocompetent individuals [1], whereas infection in utero [2] or in immunosuppressed patients after organ or stem cell transplantation [3,4] can cause severe disease. With foscarnet, ganciclovir, valganciclovir and cidofovir, several antiviral drugs targeting the viral polymerase are available for treatment or prevention of HCMV-associated disease. Their use is, however, associated with toxic side effects and emergence of resistance, limiting their application in certain situations [4,5]. Letermovir, recently approved for prophylaxis after hematopoietic stem cell transplantation, targets the viral terminase [6]. It appears to be less toxic, but the occurrence of resistance has been reported [7,8,9,10]. A relatively new field of antiviral treatment strategies is to interfere with viral entry into the target cell. Small agents and antiviral peptides are already approved against human immunodeficiency virus 1 (HIV-1) [11,12], and a peptide-based entry inhibitor is also being investigated against hepatitis B and D virus [13,14,15,16]. In cell culture, the entry of HCMV can be inhibited by a soluble derivative of its entry receptor platelet-derived growth factor receptor alpha (PDGFRα) [17,18,19], which acts as a decoy receptor by binding to the viral glycoprotein O (gO). gO is a component of the trimeric envelope complex that mediates entry into fibroblasts [20,21,22]. In addition, peptides derived from the extracellular domain of PDGFRα have also been identified that can prevent HCMV entry [18].

While the decoy receptor PDGFRα-Fc and its peptide derivatives have been shown to inhibit cell-free HCMV infection, it is unclear whether they can also impede the cell-associated growth of the virus. This is important because recent clinical isolates of HCMV spread strictly cell-associated in cell culture [23,24,25,26] and this mode of spreading is thought to be important for dissemination within a host once the infection is established [27,28,29]. Cell-associated spread is common in enveloped viruses [30], and is assumed to improve transmission efficiency by increasing the local concentration of the virus at sites of cell–cell contact. In addition, it may allow evasion of the immune system by limiting access for neutralizing antibodies in time and space. Mechanistically, this can be mediated: (i) by the adherence of progeny viruses to the surface of the producing cell; (ii) by the release of cell-free viruses into narrow gaps between adjacent cells, which can be structurally reinforced by cell adhesion molecules to form a “virologic synapse”; or (iii) by areas of cell–cell fusion through which subviral particles can be transmitted without leaving the cell. Which of these possibilities apply to HCMV is still unclear. There is some evidence for the transfer of subviral particles through fusion pores [31,32,33]. On the other hand, visualization of viral particle transfer using fluorescently labeled virion proteins supports the notion that fully enveloped particles are released from the producing cell and then enter neighboring cells [34]. Consistent with both explanations, the same glycoproteins that drive the fusion of cell-free virion envelopes with cell membranes are also essential for cell-associated spread [35]. Hence, neutralizing antibodies against glycoprotein B or glycoprotein H could, in principle, also block fusion events that mediate cell-to-cell transmission. The failure of neutralizing antibodies against cell-associated spread [34,36,37] therefore argues for transmission through a tight cell–cell contact site from which large molecules such as antibodies are excluded. Detailed analysis of the mechanisms underlying cell-associated spread of HCMV is complicated by the fact that cell-associated recent isolates are usually propagated by reseeding the infected cultures or by coculturing a few infected cells with an excess of uninfected ones [25,26,38,39,40]. This inevitably leads to asynchronous infection depending on the state of the infected cell that would transmit the virus to its uninfected neighbors. Moreover, neighboring cells are typically infected by multiple virions [34], and it is currently not possible to selectively choose the viral dose as is the case in the cell-free mode. Therefore, it is unknown whether the high multiplicity of infection during cell-associated spread is (i) a prerequisite for successful viral growth, (ii) an unnecessary excess, or (iii) a reserve that is important for the virus under conditions of an antiviral host response.

A more in-depth understanding of the processes involved in cell-associated spread of HCMV could help identify new strategies to combat this viral infection. In principle, entry inhibitors are also suitable for blocking direct cell-to-cell transmission, as shown with a small compound thiourea molecule that interacts with gB and inhibits the fusion of cell-free virions but also effectively blocks cell-to-cell spread [41,42]. PDGFRα-Fc is an entry inhibitor that binds to gO, which, like gB, is involved both in entry of cell-free HCMV and in cell-associated spread [22,35]. However, the finding that this decoy receptor failed to reduce focal growth of a laboratory strain beyond the effect of neutralizing antibody or methylcellulose overlay suggests that it was effective only against the cell-free component of this virus [19]. In the same study, antibodies against the pentameric glycoprotein complex gH/gL/pUL128/130/131A almost completely abrogated focus formation by a gO-deletion mutant. This favors the idea that gO contributes to the formation of a “virological synapse” which limits access for large molecules such as antibodies or PDGFRα-Fc. If this were the case, smaller derivatives such as the PDGFRα-derived peptides might be more effective against the cell-associated spread of HCMV. Therefore, we aimed to investigate the inhibitory effect of PDGFRα derivatives against the cell-associated growth of HCMV, which could not only evaluate their therapeutic potential but also help to gain new insights into the mechanisms underlying this mode of spread.

## 2. Materials and Methods

### 2.1. Cells and Viruses

Primary human foreskin fibroblasts (HFFs) and immortalized human fetal foreskin fibroblasts expressing the tet-repressor (HFFF-tet cells [43]) were cultivated in MEM supplemented with GlutaMax (Thermo Fisher Scientific, Waltham, MA, USA), 5% fetal bovine serum (FBS; PAN Biotech, Aidenbach, Germany), 0.5 ng/mL basic fibroblast growth factor (tebu bio, Le Perray-en-Yvelines Cedex, France) and 100 µg/mL gentamicin (Sigma-Aldrich, St. Louis, MO, USA). Experiments were performed in medium without the basic fibroblast growth factor (designated MEM5).

Low-passaged recent HCMV isolates, provided by the diagnostic laboratory of the Institute for Virology in Ulm, were propagated to infection rates of about 50% in HFFs. Cells were stored in aliquots at −80 °C. For each individual experiment, the cell-associated nature of the isolates was verified. For that, 15,000 HFFs/well were incubated in 96-well plates with the supernatant of the respective isolate overnight and fixed on the next day. Indirect immunofluorescence staining of viral immediate early (IE) antigen (Ag) was used as a readout. Isolates were considered strictly cell-associated if the number of IE Ag-positive cells per well did not exceed 10.

The HCMV strain Merlin-pAL1502 [43] and its two derivatives Merlin-pAL1502-GLuc and Merlin-pAL1502-UL32EGFP-UL100mCherry [34] were used as they resemble clinical isolates by growing strictly cell-associated in HFFs. Due to tet-operators upstream of RL13 and the UL128 locus these strains release cell-free infectivity in HFFF-tet cells that express the tet-repressor. For production of virus stocks, HFFF-tet cells were infected, supernatants were collected at 5–7 d post infection (d p.i.), clarified from cell debris by centrifugation at 2790× *g* for 10 min and stored at −80 °C until used in experiments. The obtained viral stocks were used to infect HFFs in a cell-free manner, where Merlin-pAL1502 and derivatives then spread strictly cell-associated due to lack of the tet-repressor.

The bacterial artificial chromosome (BAC)-cloned HCMV strain TB40-BAC4, which has a broad cell tropism, grows to high titers [44]. Infectious supernatants of TB40-BAC4 and its derivatives TB40-BAC4-IE-GLuc [45] and TB40-BAC4-UL74stop [46] were harvested from HFFs 5–7 d p.i. and cleared from cell debris via centrifugation at 2790× *g* for 10 min. Virions of TB40-BAC4-IE-GLuc were additionally washed twice by ultracentrifugation at 70,000× *g* for 70 min to minimize background levels of secreted luciferase. Cleared TB40-BAC4-UL74stop supernatants were 80- to 100-fold concentrated by ultracentrifugation at 70,000× *g* for 70 min. Virus stocks were stored in aliquots at −80 °C.

Infectious supernatants of the herpes simplex virus (HSV) strains R10.2 (HSV-1) and R6 (HSV-2) were harvested from infected HFFs 1–2 d p.i., cleared of cell debris via centrifugation at 2790× *g* for 10 min and stored in aliquots at −80 °C.

### 2.2. Indirect Immunofluorescence

HCMV-infected cells were fixed at room temperature (RT) with 80% acetone for 5 min and then incubated with primary antibodies for 90 min and secondary antibodies for 60 min at 37 °C. Cell nuclei were counterstained with 4′,6-diamidino-2-phenylindole (DAPI; Sigma-Aldrich, St. Louis, MO, USA) for 8 min at RT. After each incubation step, cells were washed three times with phosphate-buffered saline (PBS). Viral IE Ag was detected using monoclonal mouse antibody E13 (Argene/Biomerieux, Marcy-l’Étoile, France) and Cy3-goat-anti-mouse Ig F(ab’)_2_ (Jackson ImmunoResearch, West Grove, PA, USA) or Alexa488-goat-anti-mouse Ig F(ab’)_2_ (Thermo Fisher Scientific), resulting in a red or green nuclear fluorescent pattern. The capsid-associated tegument protein pp150 (pUL32) of viral particles was detected using monoclonal mouse antibody 36-14 [47] and Alexa488-goat-anti-mouse Ig F(ab´)_2_ or Alexa555-goat-anti-mouse Ig F(ab´)_2_ (Thermo Fisher Scientific), and this staining yielded green or red dotlike fluorescence. MCherry-tagged glycoprotein M (gM, pUL100) was detected using DsRed-specific polyclonal rabbit antibodies (Clontech, Kusatsu, Shiga Prefecture, Japan) and Cy3-goat-anti-mouse Ig F(ab´)_2_, resulting in red dotlike fluorescence.

HSV-infected cells were fixed with 4% paraformaldehyde (Sigma-Aldrich) for 10 min at RT and permeabilized with 10% sucrose (Sigma-Aldrich), 1% FBS and 0.5% Nonidet P40 (Sigma-Aldrich) for 15 min at RT. Infected cell protein 8 (ICP8) of HSV-1 infected cells was detected using monoclonal mouse antibody 11E2 (Santa Cruz Biotechnology, Dallas, TX, USA) and Alexa488-goat-anti-mouse Ig F(ab’)_2_. HSV-2 infected cells were detected using monoclonal mouse antibody 12.3.4/1.1.1 (Santa Cruz Biotechnology) and Alexa488-goat-anti-mouse Ig F(ab’)_2_.

### 2.3. Effects of Entry Inhibitors on HCMV and HSV Infection

To analyze inhibitory effects on cell-associated HCMV infection, HFFs seeded on gelatin-coated 96-well plates were infected with cell-free preparations of Merlin-pAL1502 or Merlin-pAL1502-GLuc at a multiplicity of infection (MOI) of 0.005. After 24 h, PDGFRα-Fc (R&D, Minneapolis, MN, USA) or derived individual peptides (Phtdpeptides, Shanghai, China) were added at desired concentrations and cells were cultured for a total of 7 d. For analysis of cell-associated spread of clinical isolates, infected HFF cultures were co-cultured with an excess of uninfected HFFs in 96-well plates for 7 d in the presence of PDGFRα-Fc or peptides at desired concentrations. In both assays, HCMV-IgGs (Cytotect; CP Biotest, Dreieich, Germany) at 10-fold half maximal neutralization capacity were included as a negative control. After fixation, viral IE Ag was detected by indirect immunofluorescence and the efficiency of focal spread was analyzed by counting the number of foci/well and the number of infected cells/focus.

To analyze inhibitory effects on cell-free HCMV infection, preparations of Merlin-pAL1502, TB40-BAC4, TB40-BAC4-UL74stop or TB40-BAC4-GLuc at a final MOI of 1 were preincubated with desired concentrations of PDGFRα-Fc or derived peptides for 2 h at 37 °C in MEM5. HFFs seeded on gelatin-coated 96-well plates were infected with the respective virus/inhibitor mixture for 2 h at 37 °C followed by a medium exchange with MEM5 and incubation overnight. Cells were fixed on the next day. To compare the effects on cell-free HCMV with those on HSV, preparations of HCMV TB40-BAC4, HSV-1 R10.2 or HSV-2 R6 were preincubated at a final MOI of 1 with desired concentrations of the peptide GD30 for 1 h at 37 °C in MEM5, followed by infection of HFFs for 1 h at 37 °C. The medium was replaced by MEM5, HSV-infected cells were fixed 6 h p.i. and HCMV-infected cells were fixed the next day. Cultures were stained by indirect immunofluorescence and the fraction of infected cells was determined as a readout for infection efficiency.

### 2.4. Mode of Action Analysis on the Single Particle Level

For mode of action analysis of the peptide GD30, HFFs were infected at an MOI of 0.05 with cell-free stocks of Merlin-pAL1502-UL32EGFP-UL100mCherry. GD30 was added 1 d p.i. and incubated with infected HFFs until 5 d p.i., when cultures were fixed, and viral particles were visualized by indirect immunofluorescence. HFFs in the late productive stage were identified by their strong cytoplasmic green (pp150-EGFP) and red (gM-mCherry) fluorescence, and their neighboring cells were analyzed regarding the total number of viral particles (dotlike green signals) and the number of enveloped particles (dotlike green signals that were also red fluorescent). Furthermore, the co-localization of particles with nuclei (blue fluorescence) was documented.

For analysis of recent clinical isolates, GD30 was added to freshly seeded HFF cultures in which about 5% of the cells were infected. Merlin-pAL1502-infected cultures were included as a reference. Cells were incubated for 2 d, fixed, and stained for viral IE Ag to detect infection and the viral capsid-associated tegument protein pp150 to detect virus particles. Single late stage infected cells, surrounded by newly infected neighboring cells, were documented (30 images per well) and particles were counted. The total number of particles (dotlike pp150-signals) in the surroundings of productive cells was used as a readout for adsorption of released virus progeny to neighboring cells, and the number of particles at an IE Ag-positive nucleus was used as a readout for virus penetration and translocation.

For dose–response curves on a single particle level, Merlin-pAL1502-infected HFFs were co-cultured with a 20-fold excess of uninfected HFFs and incubated with GD30 at various concentrations for 2 d. Cultures were then fixed and stained for viral IE Ag and pp150. Late stage infected cells (strong cytoplasmic pp150 signal) surrounded by freshly infected neighboring cells were identified and documented (30 frames per well). Particles located at nuclei of infected neighboring cells were counted as a readout for successful entry and translocation.

### 2.5. Synchronizing Cell-Associated Spread Using the Inhibitory Peptide GD30

HFFs seeded on gelatin-coated 96-well plates were infected with Merlin-pAL1502 at an MOI of 0.005. At 42 h p.i., medium was replaced by MEM5 for untreated virus spread or GD30 at a concentration of 125 µmol/L. The peptide was replaced 5 d p.i. by MEM5 for the desired time as indicated in text and figures. To analyze viral particle transfer and viral IE Ag expression after peptide removal in cells adjacent to productively infected cells, cultures were either fixed immediately at the end of the peptide-free period or again treated with 125 µmol/L GD30 for 5 h and stained by immunofluorescence for viral IE Ag and pp150. Cells in the direct vicinity of productive cells were evaluated for the number of virus particles that colocalized with their nuclei and their IE Ag status.

### 2.6. Statistical Analysis

Unpaired two-sided t-tests were used to identify statistically significant differences in datasets with two groups. *p*-values < 0.05 were considered marginally significant, <0.01 significant, and <0.001 highly significant. Datasets comprising more than two groups were analyzed by one-way ANOVA using the build-in data analysis functions of Microsoft Excel or SigmaPlot. If the ANOVA indicated significant differences within the dataset, a post hoc analysis was performed to compare each group to the untreated control. *p*-values were then calculated using unpaired two-sided *t*-tests.

## 3. Results

The aim of this study was to analyze the potential of PDGFRα derivatives previously found to inhibit cell-free HCMV infection with respect to inhibition of cell-associated spread. The most promising candidate should be further improved by targeted modifications and the mode of action investigated. If PDGFRα derivatives were found to inhibit cell-to-cell spread, their suitability as a tool to elucidate the mechanisms underlying this mode of spread should be investigated.

### 3.1. Cell-Associated Spread of HCMV Is Resistant to a Soluble Form of PDGFRα but Inhibited by Peptide Derivatives Thereof

PDGFRα-Fc inhibits cell-free infection of HCMV by acting as a decoy receptor that binds to the viral envelope protein gO [17,18,19], whereas its effect on cell-associated spread has not been conclusively elucidated. The findings obtained with laboratory strain TB40 suggest that this decoy receptor cannot inhibit cell-associated spread of HCMV, as it did not reduce focal size more than an overlay medium or neutralizing antibodies [19], treatments that are assumed to block cell-free virus transmission. However, confirmation of this interpretation by studying strictly cell-associated HCMV isolates was lacking. Therefore, to unambiguously investigate the effect of PDGFRα-Fc on strictly cell-associated growth, we applied it to two recent HCMV isolates or the isolate-like strain Merlin-pAL1502, which form foci of infection in HFF cultures without releasing considerable infectivity into the supernatant [24,26,43]. Merlin-pAL1502 has the advantage that it can be switched between a strictly cell-associated growth and release of cell-free infectivity by growing it in normal HFFs or HFFF-tet cells that express the tet repressor and therefore downmodulate the viral genes RL13 and UL128.

**Figure 1:**
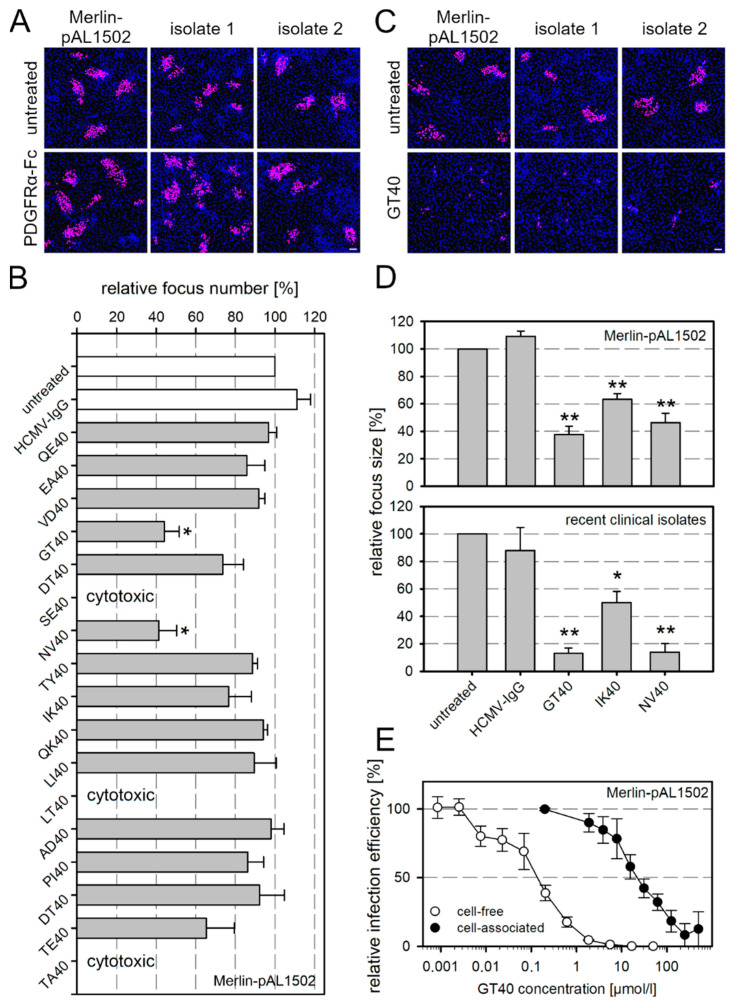
PDGFRα-derived peptides inhibit cell-associated spread of HCMV. (**A**) Fibroblasts (HFFs) infected either with the cell-associated Merlin strain pAL1502 or with recent cell-associated clinical isolates were cocultured with an excess of uninfected HFFs. Cultures were left untreated for 7 d or incubated with 250 ng/mL PDGFRα-Fc. Cells were fixed and stained for viral IE Ag via indirect immunofluorescence (pink nuclei). Nuclei were counterstained with DAPI (blue). Scale bar = 100 µm. (**B**) HFFs were infected with Merlin-pAL1502-GLuc at an MOI of 0.005. After 24 h, 40-mer derived peptides from the extracellular domain of PDGFRα were added at a concentration of 60 µmol/L. Cultures were left untreated or were incubated with HCMV-IgGs as control. Cells were fixed 7 d p.i. and stained for viral IE Ag via indirect immunofluorescence. Nuclei were counterstained with DAPI. The total focus number per well was determined (focus ≥ 3 IE Ag-positive cells) and is shown relative to the total focus number of MEM5-treated cells. Bars indicate mean values of three independent experiments, error bars represent the SEM. Asterisks indicate significant differences (*, *p*-value < 0.05). The arrangement of the peptides in the graph represents their individual location in the protein starting from the N-terminus. (**C**) HFFs infected either with the cell-associated Merlin strain pAL1502 or with recent cell-associated clinical isolates were cocultured with an excess of uninfected HFFs. Cultures were left untreated for 7 d or incubated with 60 nmol/mL of the peptide GT40. Cells were fixed and stained for viral IE Ag via indirect immunofluorescence (pink nuclei). Nuclei were counterstained with DAPI (blue). Scale bar = 100 µm. (**D**) The number of IE Ag-positive cells per focus was counted. The mean is given in comparison to the untreated controls. Bars indicate mean values of three independent experiments, error bars represent the SEM. Asterisks indicate significant differences (*, *p*-value < 0.05; **, *p*-value < 0.01). (**E**) GT40 was either incubated with cell-free preparations of Merlin-pAL1502 for 2 h prior to infection of HFFs at an MOI of 1, followed by fixation on the next day, or was added to cultures 1 d p.i. at an MOI of 0.005, and cells were fixed 7 d p.i. Cultures were stained for viral IE Ag via indirect immunofluorescence, nuclei were counterstained with DAPI. The relative infection efficiency was calculated in comparison with untreated controls. Dose–response curves show the mean values of three independent experiments, error bars represent the SEM.

The cell-associated character of the isolates was tested before each experiment by incubating uninfected HFFs overnight with cell-free supernatants of the respective isolate culture followed by immunofluorescence detection of viral IE Ag. If the number of Ag-positive cells per 15,000 indicator cells did not exceed 10, the isolate was considered cell-associated and used for subsequent experiments. HFFs containing either an isolate or Merlin-pAL1502 were reseeded with an excess of uninfected HFFs, and PDGFRα-Fc was added the day after seeding at a concentration that completely inhibited cell-free HCMV (250 ng/mL). Medium without inhibitor served as a negative control. After 7 d of co-culture, HFFs were fixed and stained for viral IE Ag by immunofluorescence. PDGFRα-Fc did neither inhibit the two clinical isolates nor Merlin-pAL1502 when compared with the untreated control (Figure 1A).

This failure of PDGFRα-Fc resembles the ineffectiveness of neutralizing antibodies in inhibiting cell-associated HCMV isolates [34,36,37]. A plausible explanation could be the limited accessibility at sites of viral transmission due to the size of the molecules. Therefore, we speculated that 40- to 50-fold smaller PDGFRα-derived peptides might be more effective to inhibit cell-associated spread. Recently, we had tested an overlapping set of 40-mer peptides derived from the extracellular domain of PDGFRα concerning inhibition of cell-free HCMV and found that three of these peptides (GT40, NV40, and IK40; Figure S1) were inhibitory at half-maximal concentrations below 10 µmol/L [18]. We now tested the same set of peptides using Merlin-pAL1502-GLuc to analyze if any of them could inhibit cell-associated spread. Peptides were added 1 d p.i. at a concentration of 60 µmol/L and cultures were incubated for 6 d. Cells were then fixed and stained for viral IE Ag by immunofluorescence. GT40 and NV40 significantly reduced the number of foci compared with the untreated control (Figure 1B,C). These two peptides and IK40 were additionally tested for activity against two recent clinical isolates and, as with Merlin-pAL1502-GLuc, significantly reduced focus size (Figure 1D). The focal growth of the isolates was reduced by more than 80% with GT40 and NV40 and by 50% with IK40. Using GT40 as an example, the inhibitory effects against cell-free and cell-associated infection were compared by generating dose–response curves for both modes of transmission. To evaluate the effect on cell-free spread, Merlin-pAL1502 preparations harvested from infected HFFF-tet cells were incubated with the peptide for 2 h before the infection of HFFs at an MOI of 1. Infected cells were incubated overnight and then fixed and stained for viral IE Ag by immunofluorescence. To analyze the effect on cell-associated spread, GT40 was added 1 d p.i. to HFFs infected with Merlin-pAL1502 at an MOI of 0.005, and then incubated for an additional 6 d. Finally, cultures were fixed and also stained for viral IE Ag by immunofluorescence. Relative infection efficiencies were calculated for both approaches compared with untreated controls. In this comparison, the half-maximal concentration for the inhibition of cell-associated spread was shifted by two log levels to the higher dose range compared with cell-free spread (Figure 1E).

### 3.2. Targeted Modifications of the Peptide GT40 Enhance Its Efficiency against Cell-Free Virus but Not against Cell-Associated Spread

**Figure 2:**
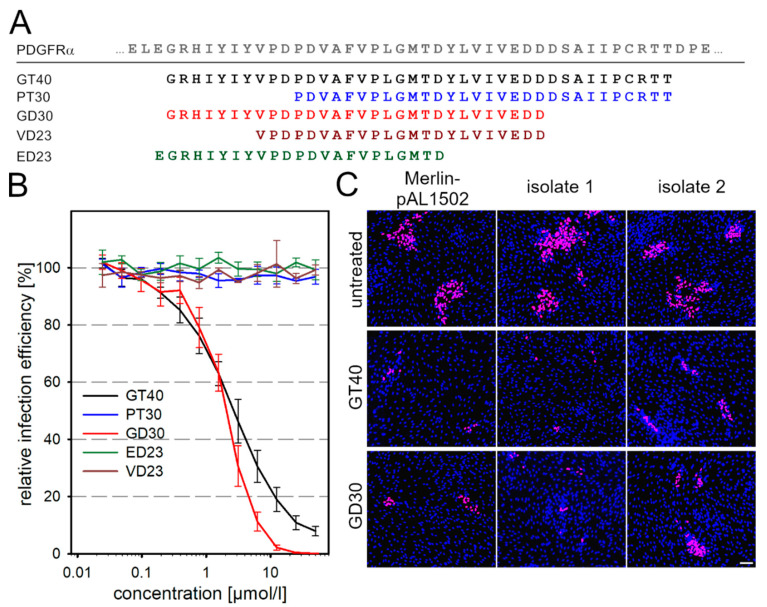
Antiviral activity of truncated GT40 derivatives against HCMV. (**A**) Amino acid sequences of PDGFRα, GT40 and its truncated derivatives. (**B**) GT40 and the four truncated peptides were incubated with cell-free TB40-BAC4 preparations for 2 h prior to infection of HFFs. Cells were incubated overnight, fixed and stained for viral IE Ag via indirect immunofluorescence. Cell nuclei were counterstained with DAPI. The relative infection efficiency was calculated in comparison with the untreated control. Dose–response curves show the mean values of three independent experiments, error bars represent the SEM. (**C**) HFFs infected either with the cell-associated Merlin strain pAL1502 or with recent cell-associated clinical isolates were cocultured with an excess of uninfected HFFs. Cultures were left untreated for 7 d or incubated with 60 µmol/L GT40 or GD30 1 d p.i. or were left untreated. Cells were fixed 7 d p.i. and stained for viral IE Ag via indirect immunofluorescence (pink nuclei). Nuclei were counterstained with DAPI (blue). Scale bar = 100 µm.

Having identified several PDGFRα-derived peptides as inhibitors of cell-associated HCMV, we aimed to investigate whether their inhibitory effects could be further enhanced by targeted modifications for potential therapeutic use. Of the three peptides that showed activity against cell-associated spread, we chose GT40 because it gave more reliable results in repeated rounds of synthesis and was more potent than IK40. Two different approaches were followed for the modifications. In the first approach, we aimed at stabilizing the conformation by cyclization via disulfide bridges. Since the original sequence of GT40 already contains a cysteine, we introduced one or three additional ones at appropriate positions to generate a total of four derivatives of GT40 that are expected to form intramolecular disulfide bonds (Table 1). In the second approach, the original peptide was truncated to further facilitate access of the molecule to the site of virus transmission, i.e., 30-mer peptides were synthesized that covered either the N- or the C-terminus of GT40 (Figure 2A).

The modified peptide derivatives were first evaluated regarding the half-maximal concentration (EC50) for inhibition of the cell-free virus. For this, virus preparations were incubated with the modified peptides for 2 h before infection of HFFs at an MOI of 1. After an overnight incubation, the cultures were fixed and stained for viral IE Ag by indirect immunofluorescence. The percentage of infected cells was determined and plotted against the peptide concentration. All cyclic derivatives had an inhibitory effect on cell-free virus but were not more effective than the original peptide GT40 (data not shown). Therefore, they were not analyzed further for potency against cell-associated spread. Among the truncated peptides, the N-terminally derived GD30 (Figure S1) was effective against cell-free virus, whereas the C-terminally derived PT30 had no antiviral function (Figure 2B). GT40 and GD30 inhibited cell-free virus with EC50 values of 2.6 and 2.1 µmol/L, respectively. The dose–response curve of GD30 was steeper than the curve of GT40 with a Hill slope of −1.67 as compared to −0.94. This resulted in a significantly stronger inhibition by GD30 at 6.25 µmol/L (*p* = 0.023), 12.5 µmol/L (*p* = 0.039), 25 µmol/L (*p* = 0.044) and 50 µmol/L (*p* = 0.041). Further truncation of GD30 to two 23-mers resulted in a loss of function (Figure 2B), indicating the importance of the two hydrophobic stretches at the N- and C-termini of GD30 in relation to its antiviral function. To address the antiviral potential of GD30 against cell-associated spread, Merlin-pAL1502 or recent clinical isolates were incubated 1 d p.i. with GD30 at a concentration of 60 µmol/L. Cultures were fixed at 7 d p.i. and stained for viral IE Ag by immunofluorescence. The 30-mer peptide reduced focal growth of Merlin-pAL1502 and recent clinical isolates to a similar extent but not more than GT40 (Figure 2C). As the same modification that increased the effect on cell-free virus did not improve the activity against cell-associated growth we wondered about the underlying mechanism.

### 3.3. GD30 Reduces the Number of Transferred Viral Particles and Their Penetration Efficiency

Regarding the mode of action of GD30 in the inhibition of cell-associated spread of HCMV, it appeared reasonable to assume that the peptide inhibits the transfer of infectious viral particles. Regarding the effect on cell-free virions, we had previously found that PDGFRα and its derived peptides inhibit the adsorption and penetration of viral particles [18,48]. By analogy, one could hypothesize that the PDGFRα derivative GD30 decreases the number of particles that can be transferred from productively infected cells to neighboring cells during cell-associated spread and/or their penetration efficiency.

**Figure 3:**
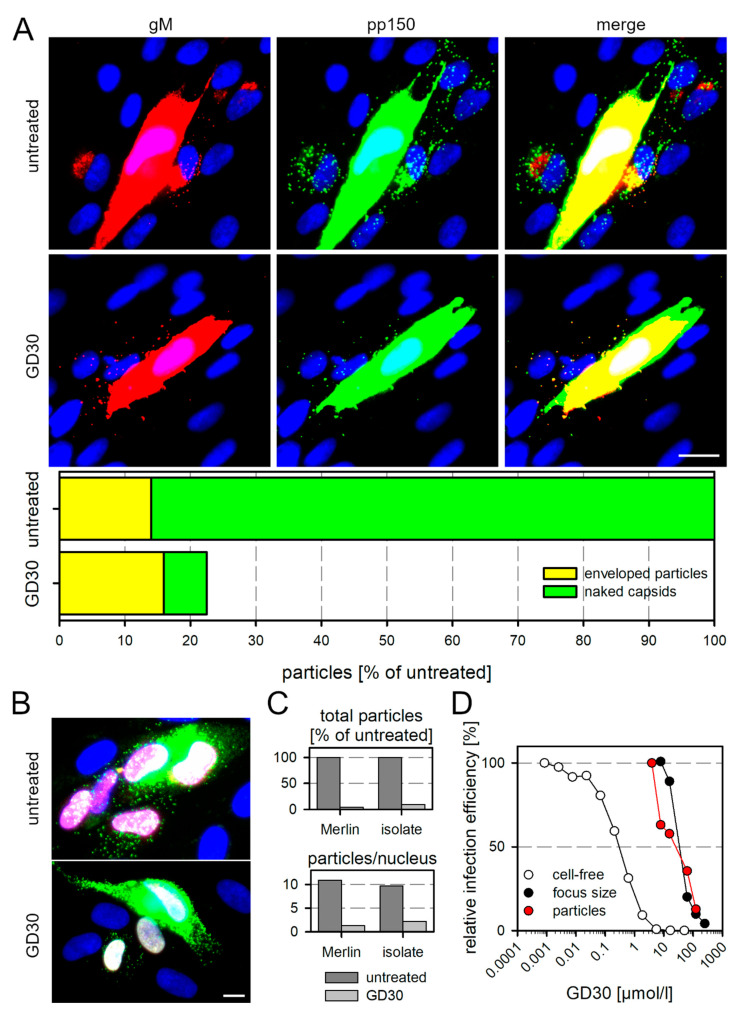
Effect of the inhibitory peptide GD30 against cell-associated spread of HCMV on the single particle level. (**A**) HFFs were infected with Merlin-pAL1502-UL32EGFP-UL100mCherry at an MOI of 0.05 and 1 d p.i. incubated with 125 µmol/L GD30 or DMSO-containing medium as untreated control. Cells were fixed 5 d p.i. and stained via indirect immunofluorescence for gM (pUL100mCherry, red) and pp150 (pUL32EGFP, green), nuclei were counterstained with DAPI (blue). For both conditions, 10 frames were documented, each with a productively infected cell in the center and viral particles in the surroundings. Examples of images used for the analysis are shown (scale bar = 20 µm). Images were analyzed for the total particle number in the surrounding of productive cells (all dotlike pp150 signals), the relative fraction of naked capsids (pp150-positive dotlike signals lacking gM) and the relative fraction of such capsids that were located at the nuclei of cells. Green bars represent naked capsids, yellow bars represent enveloped particles. (**B**) HFFs infected with recent clinical isolates were incubated with GD30 1 d p.i. and fixed 3 d p.i., when they were stained for viral IE Ag (pink nuclei) and the viral capsid-associated tegument protein pp150 (green dotlike signals). Nuclei were counterstained with DAPI (blue), scale bar = 10 µm. (**C**) The same procedure described for the clinical isolates was performed using Merlin-pAL1502. Upper panel: the total number of particles in the surrounding of productively infected cells were counted and are shown relative to the untreated control. Lower panel: only the particles that co-localized with IE Ag-positive nuclei were counted and are shown relative to the untreated control. (**D**) Merlin-infected cultures were treated with various concentrations of peptide GD30. Viral particles co-localizing with newly infected cell nuclei were counted. The numbers were plotted as percentage of untreated controls (red) and are shown in comparison to the infection efficiency of cell-free (white) and cell-associated (black) Merlin-pAL1502 in presence of the peptide.

To detect single virions that are transferred during cell-associated spread, we used a dual fluorescent labeled derivative of Merlin-pAL1502 [34] that expresses a red fluorescent protein (mCherry) fused to gM (UL100) and a green fluorescent protein (EGFP) fused to the capsid-associated tegument protein pp150 (UL32). Enveloped virions carry both the red and green fluorescent proteins and appear as yellow fluorescent dotlike signals when both channels are merged. Virions that have penetrated only appear green because they have lost the gM-containing envelope upon entry. HFFs were infected with Merlin-pAL1502-UL32EGFP-UL100mCherry at an MOI of 0.05 and incubated with the peptide GD30 at a concentration of 125 µmol/L on the next day. Medium containing DMSO at the appropriate concentration was used as a control. Cells were fixed 4 d p.i. and stained by immunofluorescence for pUL32-EGFP and pUL100-mCherry to further enhance the native fluorescence signals. Cells in the environment of productively infected HFFs were evaluated for the number of transferred viral particles and their fluorescence pattern. In the untreated control, we found up to 30 viral particles—and more on cells that were located adjacent to productively infected cells (Figure 3A), which is consistent with our previous findings [34]. Most of these particles showed only green fluorescence, indicating loss of the envelope due to penetration. In comparison, GD30 reduced the total number of particles transferred by 78% and the majority of particles still carried gM, indicating that GD30 also reduced penetration efficiency of the transferred viral particles. The percentage of naked capsids was 86% in the untreated control and only 7% with GD30.

To test whether the results obtained with the model virus Merlin truly reflect the natural situation, we also examined a recent clinical isolate at the virion level in direct comparison with the model virus. HFF cultures that contained about 5% infected cells were reseeded to start focus formation, incubated for 2 d with medium containing 125 µmol/L of the peptide GD30 or only the solvent DMSO (untreated control) and then fixed. For visualization of transferred particles, we stained the capsid-associated tegument protein pp150 using immunofluorescence. For detection of infected cells, we stained viral IE antigen also by immunofluorescence. We then evaluated the total number of punctate pp150 signals as an indicator of transferred viral particles, and the localization of particles on IE Ag-positive cell nuclei as an indicator that the particles had successfully penetrated the cell and initiated infection. Compared with the untreated control, GD30 reduced both the number of virus particles that were transferred from productively infected cells to adjacent cells and the number of IE Ag-positive cells within foci (Figure 3B). For the quantitative evaluation, we then focused only on cells expressing IE Ag. GD30 reduced both the total number of transferred particles and the number of particles that co-localized with the nucleus, indicating successful entry (Figure 3C).

Next, we were interested in seeing whether the dose–response curve regarding the inhibition of particle transfer would reflect the dose–response relationship for the effect on cell-free infection or the effect on cell-associated spread. For this we measured the reduction in particles per IE Ag-positive nucleus with increasing concentrations of GD30 and compared this with the effects of increasing GD30 concentrations on cell-free and cell-associated infection, determined as described before for peptide GT40 (see Figure 1E). As seen before with GT40, the concentration needed for inhibition of cell-associated spread was two log steps higher than for the inhibition of the cell-free virus. The dose–response curve for the reduction of particle numbers matched almost perfectly with the dose–response curve for the cell-associated spread, measured as the mean focus size (Figure 3D). Taken together, these experiments strongly suggested that the PDGFRα-derived peptide GD30 inhibits the focal growth of cell-associated HCMV strains or isolates by reducing the efficacy of particle transfer within growing foci.

### 3.4. Evidence That GD30 Specifically Targets gO of HCMV but Also a Factor Shared with Other Herpesviruses

Although the effects of GD30 on the transfer of virions in cell-associated focal growth were reminiscent of the known effect of PDGFRα derivatives on cell-free infection, the fact that 100-fold higher concentrations were required to inhibit cell-associated transfer suggested that the underlying molecular interactions between the two effects might differ.

**Figure 4:**
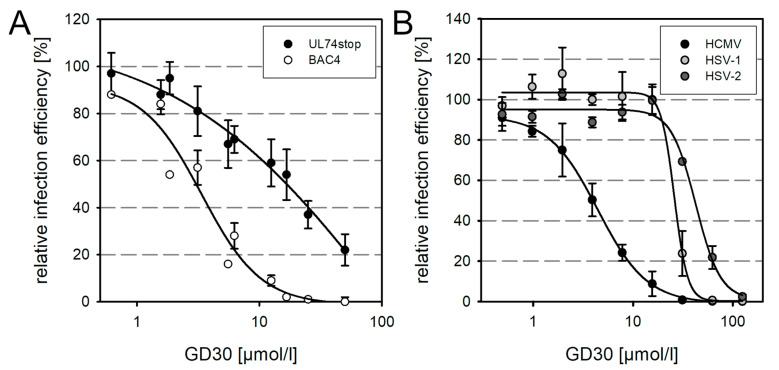
Inhibition of cell-free HCMV by GD30 is partially dependent on pUL74. The relative infection efficiency in comparison to untreated controls is plotted against the respective concentration of inhibitor. Dose–response curves represent mean values, error bars represent the SEM. (**A**) Cell-free preparations of TB40-BAC4 (final MOI of 1) and TB40-BAC4-UL74stop (final MOI > 0.3) were incubated with GD30 at desired concentrations for 2 h at 37 °C. HFFs were infected with the virus/peptide mixtures and incubated overnight. Cells were fixed and stained for viral IE Ag via indirect immunofluorescence. (**B**) Cell-free preparations of HCMV (TB40-BAC4), HSV-1 (R10.2) and HSV-2 (R6) at a final MOI of 1 were incubated with GD30 at desired concentrations for 1 h at 37 °C. HFFs were infected with the virus/inhibitor mixtures for 1 h at 37 °C. HSV-infected cells were fixed 6 h p.i., HCMV-infected cells on the next day. Cultures were stained for viral Ag via indirect immunofluorescence.

The inhibitory effect of the decoy receptor PDGFRα-Fc is based on its interaction with the gO component of the trimeric gH/gL/gO complex [17,18,19], and the selection of resistance-conferring mutations indicated that the peptide derivative GT40 also targets the trimer [48], which is also plausible considering the structural information on the interaction of the GT40-peptide with gO [49,50]. Since GD30 is merely a truncated version of GT40, interaction with gO is also conceivable as a molecular mechanism of this peptide. To test this hypothesis, we incubated cell-free preparations of HCMV strain TB40-BAC4 or a gO-deletion mutant thereof (TB40-BAC4-UL74stop) with various concentrations of GD30 for 2 h prior to infection of HFFs. On the next day, cells were fixed and stained for viral IE Ag via indirect immunofluorescence. The percentage of infected cells was determined and plotted against the peptide concentration (Figure 4A). GD30 could inhibit the TB40-BAC4-UL74stop mutant, but a 5.4-fold higher peptide concentration was required for the half-maximal effect compared with the wild-type virus. This implied that although the inhibition of the cell-free virus by GD30 is partially dependent on gO, this glycoprotein may not be the only interaction partner of the peptide. Because the inhibitory effect of the peptide against cell-free virus did not appear to be exclusively gO-dependent, we were interested in whether it was HCMV-specific or whether it might also act against other herpesviruses. Cell-free preparations of HCMV and HSV-1 and -2 were incubated with GD30 for 1 h prior to infection of HFFs at an MOI of 1. Because HSV has a faster replication cycle compared to HCMV, HSV-infected cells were fixed 6 h p.i., whereas HCMV-infected cells were fixed on the next day. Cultures were stained via indirect immunofluorescence for viral antigens. The percentage of infected cells was determined and plotted against the respective concentration of GD30 (Figure 4B). The peptide inhibited both HSV-1 and HSV-2 completely. However, compared to HCMV, there was a 5.9-fold and 9.6-fold shift in EC50 values toward the higher dose range, respectively. The efficacy of GD30 against HSV further strengthens the assumption that there is another interaction partner besides gO. Considering the concentration needed for inhibition of cell-associated spread, it appears plausible that this effect is rather mediated by the second, as-yet undefined, interaction.

### 3.5. GD30 Inhibits Cell-Associated Spread of HCMV in a Reversible Manner

**Figure 5:**
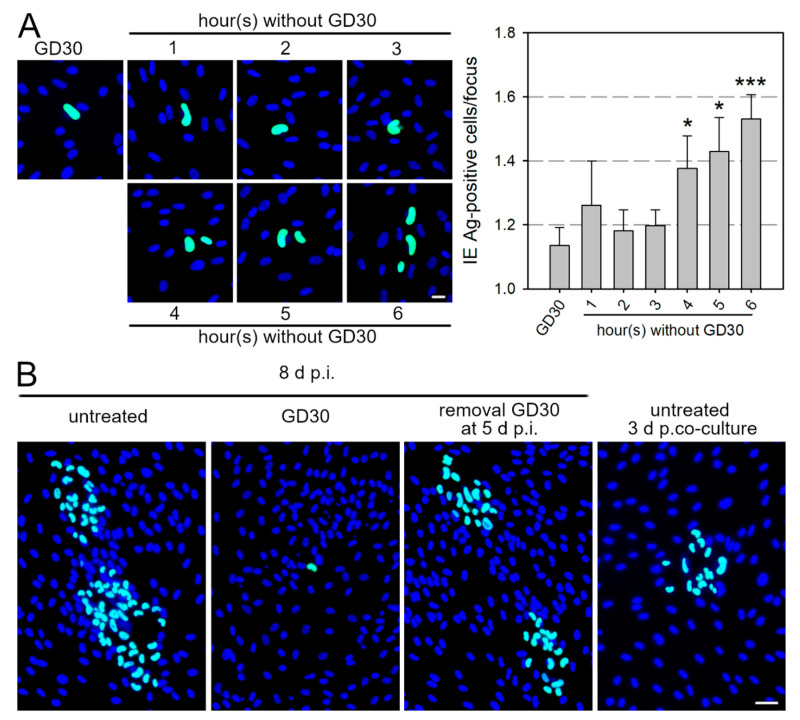
The inhibitory peptide GD30 inhibits cell-associated spread of HCMV in a reversible manner. HFFs infected with Merlin-pAL1502 at an MOI of 0.05 were incubated with 125 µmol/L GD30 or were left untreated. (**A**) At 5 d p.i., GD30 was replaced by MEM5 for time frames of 1 up to 6 h. Cells were fixed after the peptide-free period and stained for viral IE Ag by indirect immunofluorescence (green nuclei). Nuclei were counterstained with DAPI (blue). Examples of images used for the analysis are shown (scale bar = 20 µm). The number of infected cells per focus was determined. Bars indicate mean values of six independent experiments (approximately 740 foci per condition), error bars represent the SEM. Asterisks indicate significant differences (*, *p*-value < 0.05; ***, *p*-value < 0.001). (**B**) Untreated Merlin-pAL1502-infected HFFs were co-cultured with uninfected HFFs at 5 d p.i. At 8 d p.i., cultures were fixed and stained for viral IE Ag via indirect immunofluorescence (green nuclei). Nuclei were counterstained with DAPI (blue), scale bar = 50 µm.

Although the molecular mode of action of GD30 has not been fully elucidated, its identification as an inhibitor of cell-associated spread offers additional opportunities besides therapeutic application. We wanted to investigate whether this inhibition is reversible, i.e., whether the effect lasts only as long as the inhibitory peptide is present in the cell culture. This would allow controlled, synchronized, and metered particle transfer during cell-associated spread, because productively infected cells could transfer virus particles to their neighboring cells only after removal of the peptide. To investigate this issue, HFFs were infected with Merlin-pAL1502 at an MOI of 0.005, and after 42 h p.i., GD30 was applied at a concentration of 125 µmol/L, which almost completely inhibits cell-associated spread. This time point for the addition of GD30 was chosen because initial experiments had shown that the production of viral progeny within infected cells had already begun, but virions were not yet detectable on adjacent cells. After 5 d p.i., the peptide solution was replaced with MEM5 for time frames ranging from 1 to 6 h. Cells were fixed and stained for viral IE Ag by immunofluorescence. In cultures permanently treated with GD30, almost exclusively single infected cells were detected (Figure 5A). The cultures in which the peptide had been removed for increasing time frames showed an increase in infected cells/focus over time. While no difference in focus size was observed until 3 h after removal of GD30, significantly more infected cells per focus were detected after 4 and 5 h compared to permanent peptide treatment (*p*-values < 0.05), and after 6 h the increase was highly significant (*p*-value < 0.001) (Figure 5A). We next wanted to know whether the inhibitory effect of GD30 is fully reversible, i.e., whether focus size in cell cultures after removal of GD30 equals that of infected cultures that are allowed to form foci for the same time interval. For that reason, HFFs were again infected with Merlin-pAL1502 at an MOI of 0.005, 125 µmol/L GD30 were added at 42 h p.i. and withdrawn at 5 d p.i., and then incubated for further 3 d. For comparison, infected HFFs that had not been treated with GD30 were freshly co-cultured with uninfected HFFs and also incubated for 3 d. At 8 d p.i., cells were fixed and stained for viral IE Ag via indirect immunofluorescence. To control for the initial inhibitory effect of GD30, the virus was allowed to spread over the complete period of 8 d without intermittent GD30 treatment. To control for the full effect of GD30, the peptide was applied over the complete period from 42 h p.i. until fixation at day 8. In these permanently GD30-treated HFFs, almost exclusively single infected cells were detected, whereas foci with up to 50 infected cells were detected in the untreated control (Figure 5B). As expected, fewer infected cells per focus were detected in cultures that were intermittently treated with GD30 from 42 h to 5 d p.i. The mean focus size in these cultures was around 15 infected cells per focus, which resembles the focal growth after 3 d in the untreated cocultures that had been freshly seeded at 5 d p.i. Thus, the inhibitory effect of GD30 appears to be completely reversible.

### 3.6. GD30 Can Be Used to Study Underlying Mechanisms of Cell-Associated Spread

**Figure 6:**
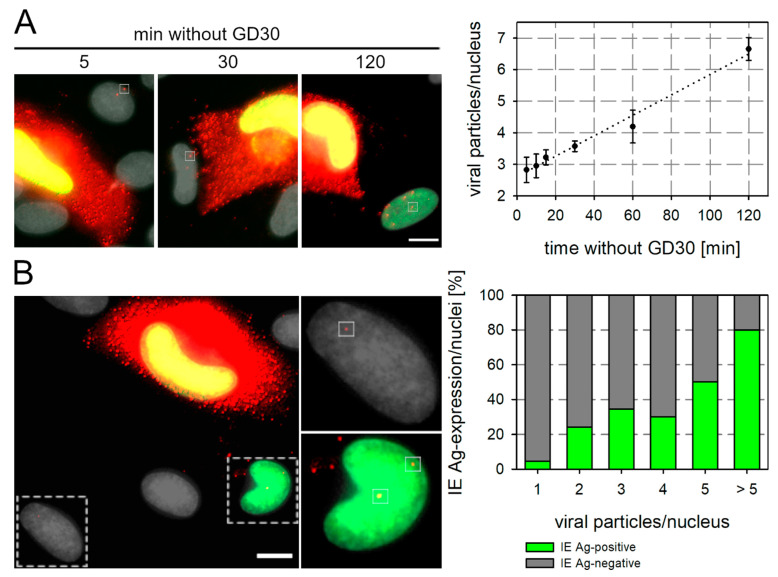
Cell-associated spread proceeds in a linear fashion and a few viral particles are already sufficient for infection of adjacent cells. HFFs infected with Merlin-pAL1502 at an MOI of 0.05 were incubated with 125 µmol/L GD30. (**A**) At 5 d p.i., GD30 was replaced by MEM5 for time frames of 5, 10, 15, 30, 60 and 120 min. Cells were fixed after the peptide-free period and stained for viral IE Ag by indirect immunofluorescence (green nuclei) and pp150 (red dotlike signals) via indirect immunofluorescence. Nuclei were counterstained with DAPI (gray). Examples of images used for the analysis are shown and one virus particle is framed as an example in each image (scale bar = 10 µm). The mean number of particles per nucleus of cells adjacent to productively infected cells is plotted against the incubation time without GD30. Values indicate the mean of six independent experiments (surrounding of approximately 60 productively infected cells per time point), error bars represent the SEM. Linear regression is equal to y = 0.03 + 2.6x. (**B**) At 5 d p.i., GD30 was replaced by MEM5 for a short time frame before the culture was again incubated with 125 µmol/L GD30 for 5 h. Cells were fixed and stained via indirect immunofluorescence for viral IE Ag (green nuclei) and the viral capsid-associated tegument protein pp150 (red dotlike signals). Nuclei were counterstained with DAPI (gray). In six independent experiments, 90 nuclei with one, two, three, four, five, or more than five colocalized particles were identified in the vicinity of productively infected cells and analyzed for their IE-Ag status. An image used for analysis with one or two virus particles per nucleus is shown as an example, virus particles are framed. Scale bar = 10 µm.

At the IE Ag expression level, the reversibility of the inhibition by GD30 became detectable as early as 4 h (see Figure 5A). This suggests that the transfer of viral particles occurs after minutes or a few hours upon peptide withdrawal, since the viral particles must first be transferred to neighboring cells, where they adsorb and penetrate. Then, the viral genome is transported into the nucleus, transcribed into mRNA, which is subsequently transferred into the cytoplasm, where it is finally translated into viral proteins. Thus, subtracting the time required for all these processes from our delay of 4 h, not much time can elapse before particle transfer occurs after peptide withdrawal. To actually determine how many particles are transferred and how fast this occurs, HFFs were infected with Merlin-pAL1502 at an MOI of 0.005 and treated with 125 µmol/L GD30 at 42 h p.i. GD30 was removed from cells 5 d p.i. for 5, 10, 15, 30, 60, and 120 min, and the cells were fixed immediately after the time expired. The cultures were then stained for viral IE Ag and pp150. Productively infected cells were imaged and cells in the direct neighborhood were examined for the detection of pp150 dotlike signals on their nuclei. Virus particles were detected on the nuclei of neighboring cells as early as 5 min after the removal of GD30 (Figure 6A), confirming that the effect of the peptide is immediately reversible. The mean number of particles per nucleus increased consistently over time by an average of 1.8 viral particles per hour. Next, we wanted to investigate whether the high MOI of 30–50 virus particles is an excess or whether it is a prerequisite for successful infection of neighboring cells. Therefore, HFFs were infected with Merlin-pAL1502 and incubated with GD30 at a concentration of 125 µmol/L at 42 h p.i. At 5 d p.i., GD30 was replaced with MEM5 for defined periods between 15 and 120 min to allow transfer of a few virus particles. GD30 was then added to the cultures for an additional 5 h, as the previous data indicated that viral IE can be detected 4–5 h after particle transfer and expression should be captured in cells which became infected during the peptide-free time. HFFs were fixed and stained for viral IE Ag and pp150 via immunofluorescence. Cells in the surroundings of productively infected cells were analyzed regarding the number of viral particles on their nuclei and expression of IE Ag. Based on the assumption that only a few particles might be sufficient for infection, cells were categorized as having one to five or more particles located on the nucleus, indicating entry and translocation to the nucleus. Of the cells with one, two or three virus particles per nucleus, 5%, 25% or 35% were IE Ag-positive (Figure 6B). This low dose threshold indicates that as few as one, two or three particles are already sufficient for successful infection of adjacent cells. In summary, these experiments demonstrate that GD30 is suitable to achieve synchronized and dosed infections of cell-associated spread and allows us to study the underlying mechanisms of this virus transmission.

## 4. Discussion

By screening an overlapping peptide set derived from the extracellular domain of the cellular HCMV receptor PDGFRα, we identified 40-mer peptides and a 30-mer that can block cell-associated spread of HCMV by reducing particle transfer from infected to uninfected cells. These inhibitors may, on the one hand, provide a new anti-HCMV treatment strategy and, on the other hand, might be a helpful tool to lead to a better understanding of the mechanisms underlying cell-associated spread.

Many viruses, including herpesviruses, can spread by two modes of transmission [51]. Infectious virions can either be released from the cell and reach new target cells by diffusion in the extracellular fluid, or they can be transmitted directly from a productively infected cell to adjacent, yet uninfected, cells [30]. Cell-associated spread is considered advantageous for the virus concerning several aspects, including an increased local concentration of infectious progeny virions and the exclusion of antibodies at zones of tight cell–cell-contacts. In principle such “cell-associated” spread could be mediated by the transmission of subviral non-enveloped particles through intercellular fusion pores [52]. However, experimental data for most viruses tend to support mechanisms involving fully enveloped extracellular particles [53]. This distinction is relevant when it is attempted to inhibit cell-associated transmission by antiviral agents that target the entry process.

With regard to HCMV, there are hints at the formation of fusion pores between infected cells and their uninfected neighbors [31,32], but data obtained with a fluorescent-tagged cell-associated version of strain Merlin indicate that fully enveloped virions are transmitted during focal spread of this virus [34]. As the same glycoproteins that mediate cell-free infection of HCMV are also involved in cell-associated spread [35], it was tempting to speculate that this transmission mode can be targeted by entry inhibitors. This assumption is supported by our finding that certain peptides derived from the extracellular domain of the HCMV receptor PDGFRα, which block the adsorption and penetration of cell-free virions [48], also inhibit the cell-associated spread of clinical HCMV isolates and the cell-associated model virus Merlin. This effect was mediated by reducing the number and the penetration efficiency of transferred viral particles, thus resembling the effect on the cell-free virus.

Our finding that the full-length entry inhibitor PDGFRα-Fc failed to reduce growth of cell-associated recent HCMV isolates, whereas 40-mer peptides thereof were effective, indicates that large molecules may be excluded from sites of cell-to-cell transmission. This is in line with failure of PDGFRα-Fc to restrict the cell-associated spread of a laboratory strain further than a methylcellulose overlay [19]. It is also consistent with the inefficacy of neutralizing antibodies against the focal growth of recent HCMV isolates in fibroblast cultures [34,36] and direct cell–cell transmission of strain Merlin [37]. Beside simple size considerations, the time available for an entry inhibitor to block cell-associated spread may also play a role. Our particle transfer kinetics after GD30 withdrawal indicate that the interval between release of virus progeny and entry into the adjacent target cell is in the range of minutes. In contrast, the incubation time in a neutralization assay is typically several hours. If the time window for inhibition is much shorter in the cell-associated mode, this could also explain why higher peptide concentrations were needed for the inhibition of cell-associated spread as compared to cell-free spread.

Fibroblast-adapted HCMV strains that spread efficiently via the cell-free route also form small foci of infected cells in the presence of neutralizing antibodies, and this antibody-resistance is sometimes used to define cell-associated transmission of these strains [33,36,54,55]. This appears appropriate as other approaches to capture cell-free HCMV, e.g., by agarose or methyl cellulose overlays result in a similar focus size, cannot be further reduced by neutralizing antibodies [19,56]. However, data from other viruses, such as HIV or HSV, indicate that cell-associated spread is not necessarily antibody-resistant [57,58,59,60]. Some broadly neutralizing antibodies targeting the interaction between the HIV envelope protein on the surface of infected cells and CD4 on target cells can prevent the formation of the virological synapse and subsequently reduce cell-associated spread [61]. Based on this finding, it is tempting to speculate about an analogous mechanism in HCMV. It is conceivable that a similar interaction between gO and PDGFRα promotes the formation of a “virological synapse” between productively infected fibroblasts and adjacent uninfected cells. In this case, antibodies against gO might render cell-associated transmission in HFFs sensitive to neutralizing antibodies by preventing tight cell–cell-contacts that would limit the access for antibodies. In that context it is noteworthy that cell-associated growth of certain HCMV isolates and mutants but (not the strain Merlin) was reported to be more sensitive to neutralizing antibodies in endo- and epithelial cells [34,37,62,63,64], which do not express PDGFRα. Apart from this speculation, an in-depth investigation of cell–cell-interactions during cell-associated spread may reveal new targets for anti-HCMV strategies.

Regarding the mechanism of action of the PDGFRα-derived peptide GD30, it is plausible to assume a priori that it disrupts the interaction of gO and PDGFRα, as shown for the full-length decoy receptor PDGFRα-Fc [17,18,19]. In line with this assumption, GD30 reduced the number of particles that were transferred from late stage infected cells to their uninfected neighbors, and the majority of these particles were not penetrated. This resembles the effect of PDGFRα-Fc and its peptide derivatives on the adsorption and penetration of free virions [18,48]. The inhibition of cell-free infection by GD30 was HCMV-specific and depended on the expression of gO, which further supports the idea that peptides and full-length proteins share the same mode of action. In addition, the same PDGFRα-derived peptides that were previously identified as inhibitors of cell-free infection [18] have now been found to be effective against cell-associated HCMV, suggesting a common mode of action. However, a caveat comes from the finding that far higher concentrations of GD30 were necessary to inhibit cell-associated spread as compared to cell-free infection. Part of this difference might be due to: (i) the high infection multiplicity in cell-associated spread; (ii) the time course of direct cell-to-cell transmission that is presumably shorter than incubation in our cell-free inhibition assays; and (iii) the possibility that even for a 30-mer peptide access to the transmission site may be impeded. Nevertheless, we have doubts about whether this can explain a 100-fold shift in the half-maximal effective concentration. These doubts are further substantiated as GD30 also inhibited a gO-deletion mutant of the TB40/E strain and herpes simplex virus at concentrations that were necessary to reduce cell-associated spread, which hints at a second mode of action at higher concentrations. At present we can only speculate about the nature of this mechanism. It is noteworthy that PDGFRα has been reported to bind gB when it is expressed at high levels [19,65], and it is conceivable that, vice versa, high levels of PDGFRα-derived peptides may bind to gB and block its fusogenic activity, as shown for a small compound cytomegalovirus fusion inhibitor, CFI02 [41,42]. In principle, an effect of GD30 on the capacity of the infected cell to produce virions also deserves consideration. However, it is unlikely that GD30 primarily acts by an effect on the producer cell because the peptide did not appear cytotoxic in a metabolic activity assay (Figure S2) and its effects were fully reversible immediately after withdrawal. If infected cells were generally reduced in virus production, one would expect a need for recovery time and an increase of the particle transfer rate after such a recovery. Instead, particle transfer started immediately after removal of GD30 at a transfer rate that remained constant over time, arguing for an effect on the virus particles rather than on the cells.

Concerning the potential of PDGFRα-derived peptides as a novel option for the development of anti-HCMV treatment strategies, our analysis provides at least proof of principle that the near complete inhibition of cell-associated infection by interference with particle transmission is possible. Attempts to facilitate the diffusion of the original GT40 peptide to transmission sites by truncation did not improve the potency against cell-associated spread, although the efficacy against cell-free viruses was slightly increased. This does not preclude the possibility that other modifications may be more successful. For example, the efficacy of the peptide-based HIV entry inhibitor T20 was greatly improved by the targeted replacement of individual amino acids or the addition of membrane anchors [66]. For a targeted improvement of GD30, the unequivocal identification of the target structure on the viral or cellular surface, as discussed before, is an important prerequisite that should be addressed next.

While further improvement of GD30 is certainly desired for its development as a therapeutic entry inhibitor, its properties qualify it as a tool for studying basic aspects of cell-associated spread. The cell-associated transmission of progeny virions can be effectively blocked, and this effect is fully reversible upon the removal of the peptide, which allows synchronized infections with cell-associated HCMV isolates. By readdition of the peptide after an appropriately selected time interval, the number of virus particles transferred can be adjusted very precisely. As a proof of concept, we applied this approach to measure the rate of particle transfer in a growing focus of the cell-associated model virus Merlin-pAL1502 and to determine the minimal number of virions that are required to initiate viral gene expression. We found that about two particles are transferred per hour from a productively infected cell towards its uninfected neighbors, which provides an explanation for the high multiplicity of infections that we had previously found when we analyzed particle numbers in untreated foci of this strain [34]. Our dose–response analysis using various peptide-free time windows revealed that in principle one particle is sufficient for infection. Therefore, an infection multiplicity of up to 50 particles per cell in recent clinical isolates appears unreasonably excessive at a first glance. However, the efficacy of viral immediate early gene expression increases in a dose-dependent manner, which means that the virus will most probably accelerate its replication cycle by increasing the infection multiplicity. This might be advantageous in the presence of antiviral host defense by reducing the time until viral immune evasion genes are expressed. Additionally, it is conceivable that such a high virus dose is necessary for HCMV in vivo to overcome cellular restriction factors that are upregulated by interferons [67,68,69]. Consistent with this, cell-associated spread has recently been reported to be more resistant to interferon-induced antiviral factors in comparison to cell-free spread [37].

In summary, this work identifies PDGFRα-derived peptides as novel inhibitors of cell-associated HCMV and demonstrates their potential as a research tool to further investigate this mode of spread.

## Figures and Tables

**Table 1:** Cyclic derivatives of GT40.

**GT40**	GRHIYIYVPDPDVAFVPLGMTDYLVIVEDDDSAIIP**C**RTT
**GT40/G1C_cyc**	**C**RHIYIYVPDPDVAFVPLGMTDYLVIVEDDDSAIIP**C**RTT
**GT40/G1C/D30C/C37S_cyc**	**C**RHIYIYVPDPDVAFVPLGMTDYLVIVED**C**DSAIIPSRTT
**GT40/P9C/S32C/C37S_cyc**	GRHIYIYV**C**DPDVAFVPLGMTDYLVIVEDDD**C**AIIPSRTT
**LT-53_cyc**	T**C**YYNHTQTEENELEGRHIYIYVPDPDVAFVPLGMTDYLVIVEDDDSAIIP**C**R

## Data Availability

Not applicable.

## Supplementary Materials

The following are available online at www.mdpi.com/1999-4915/13/9/1780/s1, Figure S1: Localization of the HCMV inhibitory peptides IK40, NV40, GT40 and GD30 in the extracellular domain of human PDGFRα. Figure S2: The peptide GD30 does not affect cell viability.
