# Supplementary material for: Peptide Derivatives of Platelet-Derived Growth Factor Receptor Alpha Inhibit Cell-Associated Spread of Human Cytomegalovirus
Source: Viruses. 2021 Sep 6;13(9):1780. doi: 10.3390/v13091780 (PMC8473290; doi:10.3390/v13091780)
Supplement: Supplementary file 1 [file viruses-13-01780-s001.zip › viruses-1340725-supplementary.pdf]

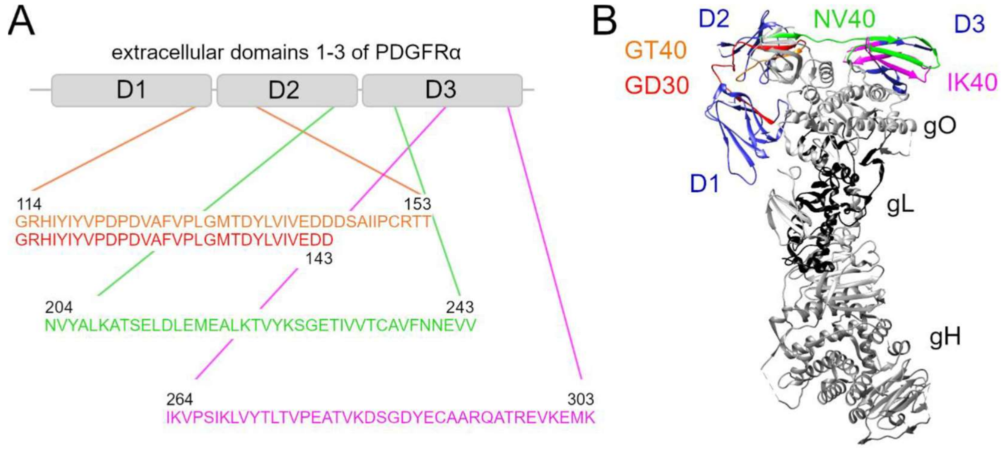

Figure S1. Localization of the HCMV inhibitory peptides IK40, NV40, GT40 and GD30 in the extracellular domain of human PDGFRα. (**A**) Schematic organization of domains 1-3 of human PDGFRα. Amino acid sequences of the HCMV inhibitory peptides IK40, NV40, GT40 and GD30 are specified. (**B**) For visualization of the peptides IK40, NV40, GT40 and GD30, the cryoEM structure of the trimeric HCMV gH/gL/gO complex bound to human PDGFRα and neutralizing fabs 13H11 and MSL-109 was used (PDB:7LBF [1]). The image was created with UCSF Chimera, a software from the Resource for Biocomputing, Visualization, and Informatics at the University of California, San Francisco [2]. Positions of IK40, NV40, GT40 and GD30 in PDGFRα domains 1-3 (D1-3) are highlighted. Those parts of the PDGFRα domains that are not covered by peptides appear in blue color.

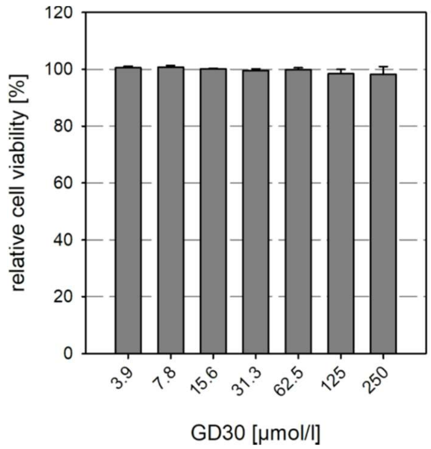

Figure S2. The peptide GD30 does not affect cell viability. HFFs were treated with GD30 at desired concentrations at 42 h p. seeding. CellTiter-Blue reagent was added at 5 d p. seeding for 3 h at 37 °C. Fluorescence was measured at 560/590nm and relative cell viability was calculated as the ratio of treatment with GD30 compared to DMSO containing medium. Bars indicate the mean of four independent experiments, error bars represent the SEM.

References

1. Kschonsak, M., Rougé, L., Arthur, C.P., Hoangdung, H., Patel, N., Kim, I., Johnson, M.C., Kraft, E., Rohou, A.L., Gill, A., Martinez-Martin, N., Payandeh, J., Ciferri, C., 2021. Structures of HCMV Trimer reveal the basis for receptor recognition and cell entry. Cell 184, 1232-1244.e16. https://doi.org/10.1016/j.cell.2021.01.036
2. Pettersen, E.F., Goddard, T.D., Huang, C.C., Couch, G.S., Greenblatt, D.M., Meng, E.C., Ferrin, T.E., 2004. UCSF Chimera--a visualization system for exploratory research and analysis. J Comput Chem 25, 1605–1612. https://doi.org/10.1002/jcc.20084
